# Geographic Profiling of *Aspergillus* Species and Aflatoxin Variants Across Peanut-Growing Regions of Queensland Australia

**DOI:** 10.3390/jof12070463

**Published:** 2026-06-24

**Authors:** Rebecca Payne, Dante L. Adorada, Graeme C. Wright, Surya Bhattarai

**Affiliations:** 1 Centre for Crop Health, School of Science, Engineering and Digital Technologies, University of Southern Queensland, Toowoomba, QLD 4350, Australia; dante.adorada@unisq.edu.au (D.L.A.); wrightgraeme@hotmail.com (G.C.W.); surya.bhattarai@unisq.edu.au (S.B.); 2 School of Health, Medical and Applied Sciences, Central Queensland University, Rockhampton, QLD 4701, Australia

**Keywords:** *Aspergillus flavus*, *Aspergillus parasiticus*, groundnut

## Abstract

Aflatoxins are carcinogenic secondary metabolites produced by *Aspergillus flavus* and *Aspergillus parasiticus*. These two fungi are ubiquitous in soil and are often found in agricultural fields. Four aflatoxin variants commonly found in infected crops are: AFB_1_, AFB_2_, AFG_1_, and AFG_2_. *Aspergillus parasiticus* can produce all aflatoxin variants, with *A. flavus* only able to produce the aflatoxin B variants. Production of aflatoxins typically occurs as pre-harvest contamination in the Australian peanut-growing regions of Queensland. This study analysed geographic variations in aflatoxin component variants using the HPLC method for the 2020–2024 season peanuts. Aflatoxin-G was found as most common aflatoxin variant across three of the four peanut-growing regions. This study also evaluated diversity of *A. flavus* and *A. parasiticus* across the four peanut-growing regions, using post-harvest soil samples from the 2023–2024 growing season. *Aspergillus parasiticus* was found to be most prevalent (97% isolates) across the regions, whereas *A. flavus* was least prevalent (3% isolates) and only found in the Tolga region. The North Burnett had no *Aspergillus* colonies identified from the soil samples in the current year of collection. The data suggests the aflatoxin G variant is most predominant in Australian peanuts and also that there is large variation between the growing regions for prevalence of *Aspergillus* species.

## 1. Introduction

*Aspergillus* section Flavi are a group of filamentous fungi which is ubiquitous in the soil [1,2]. These fungal species can produce various mycotoxins, with aflatoxin being the most severe to human health [3]. *Aspergillus flavus* and *Aspergillus parasiticus* are the most common aflatoxin-producing species among Australian peanuts [4]. Pitt and Hocking [4] also reported that *A. parasiticus* is the dominant species within Australian peanut fields; however, their data is unpublished. *Aspergillus flavus* is typically able to produce AFB_1_ and AFB_2_ aflatoxin variants and *A. parasiticus* can produce AFB_1_, AFB_2_, AFG_1_, and AFG_2_ variants [5,6,7]. These four aflatoxin variants have been declared Group 1 carcinogens by the International Agency for Research on Cancer [3].

Aflatoxin contamination typically occurs due to pre-harvest contamination in Australia. When peanuts are grown under adequate rainfall and/or irrigation, aflatoxin production rarely occurs [8]. Drought and moisture stress can cause microscopic fissures in the seed coat, along with poor pod filling and development, which results in shrivelled seeds and cracks that allow fungal entry into the peanuts [9,10]. Drought stress also reduces water activity in the soil and peanut kernels, which reduces the growth and activity of bacteria, amoebae and competing fungi which then promotes the growth of both *A. flavus* and *A. parasiticus* [8]. Within Australia, peanuts are grown under conditions favourable to aflatoxin production. A large proportion of Australian peanuts is grown under rainfed conditions, which generally results in end-of-season drought and high temperatures during the pod filling period conducive for aflatoxin growth [4].

Within Australia, peanuts are mostly grown in Queensland (QLD) with four main growing regions. These are the Atherton Tablelands in north QLD, Wide Bay-Burnett, North Burnett, and South Burnett in south QLD. These four regions have varying climates and soil compositions, with soil variations also present within each region. There have been previous studies showing that the distribution, toxin-producing capacity and contamination of *Aspergillus* spp. in peanut vary between different soil types [11]. Due to the variability of *Aspergillus* spp. and aflatoxin production among different soil types and geographic locations, as well as the unanswered question of whether *A. parasiticus* is the dominant *Aspergillus* species within Australia, a geographic profile of the *Aspergillus* and aflatoxin variants is required.

This research aimed to evaluate and identify the variation in the aflatoxin component variants from aflatoxin-positive peanut samples collected from the peanut-growing seasons of 2020–2024, along with the *Aspergillus* species distribution from soil samples collected immediately following the 2023/2024 peanut-growing season across the four main peanut-growing regions of QLD, Australia.

## 2. Materials and Methods

### 2.1. Long-Term Peanut Aflatoxin Data

High Performance Liquid Chromatography (HPLC) data were provided by the Peanut Company of Australia (PCA) (Kingaroy, Australia). The HPLC data contains aflatoxin contamination from peanuts at peanut intake for peanut-growing seasons from 2020 to 2024. After farmers harvested and dried peanuts on the farm, the products were taken to peanut shelling companies for further processing. During the intake process, peanuts were sampled from each truck load and tested for initial aflatoxin contamination and moisture content to determine segregation of the peanuts for storage. These samples were then sent to the PCA technical lab for further analysis, including HPLC analysis for aflatoxin component variants, namely AFB_1_, AFB_2_, AFG_1_ and AFG_2_.

### 2.2. Soil Sampling

**Figure 1:**
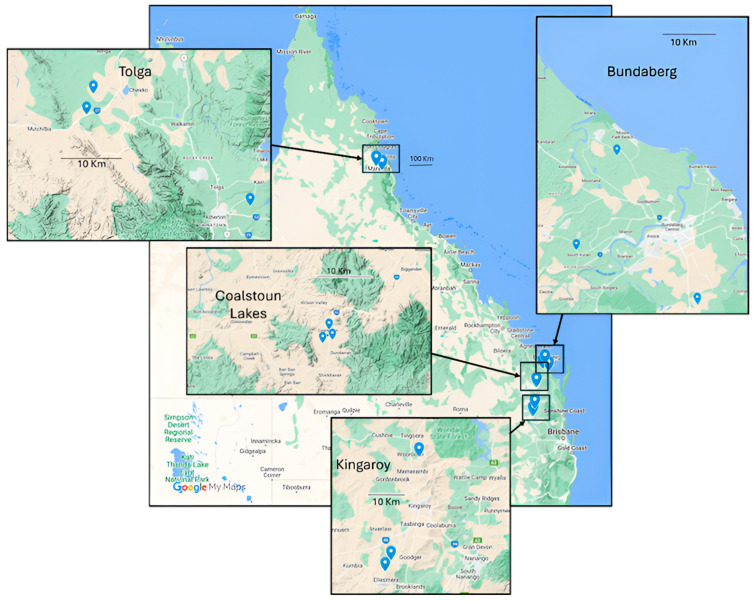
Map of locations of sites selected for soil sampling: Tolga, Bundaberg, North Burnett (Coalstoun Lakes), and South Burnett (Kingaroy) [1].

Four peanut-farming locations across QLD, Australia, were selected for this study (Figure 1). These locations produce most of the peanuts grown within Australia. Each location had three fields sampled, each containing peanuts before soil collection. These sampling locations have varying environments, with variation in elevation, climate, rainfall, and temperature (Table 1). Soil samples were collected in a zig-zag pattern in a farm plot that had a recent peanut crop present in the current growing season. The locations sampled are shown in Figure 1, indicated by the blue markers. Three aggregate samples were collected from thirteen sample points collected at 10–15 cm depth. Soil was kept at 4 °C until ready for further analysis.

### 2.3. Preparation of Media

#### 2.3.1. *Aspergillus flavus* and *parasiticus* Agar (AFPA)

To prepare the media, the following were added in a litre of distilled water: 20 g yeast extract, 10 g bacterial peptone, 0.5 g ferric ammonium citrate, 0.1 g chloramphenicol, 0.1 g dichloran, and 15 g agar. The mixture was shaken until all reagents were suspended. The media was sterilised by autoclaving at 121 °C and 15 psi for 20 min. Afterwards, it was allowed to cool to 50 °C and poured into 9 cm Petri dishes, then stored at 4 °C and used within 7 days.

#### 2.3.2. Coconut Cream Agar (CCA) Media

To produce a 50% coconut cream agar, 500 mL of coconut cream, 500 mL of distilled water and 15 g agar were thoroughly mixed together. The media was sterilised at 121 °C and 15 psi for 20 min, then allowed to cool to 50 °C before pouring into 9 cm Petri dishes stored at 4 °C and used within 7 days.

### 2.4. Plating Soil Samples on AFPA

In a 50 mL Falcon tube, 45 mL of sterile water and 5 g of soil were added to create a stock sample (100). Five millilitres were taken from the first tube and serially diluted until a 10^−5^ dilution was reached. From each dilution, 100 μL was added to the AFPA plates and spread evenly. Plates were sealed with parafilm and incubated at 30 °C for 3–5 days. Isolates of *A. flavus* and *A. parasiticus* showed a bright orange colour on the reverse colonies. Each soil sample was sampled in duplicate.

Colony forming units (CFU) were calculated for the colonies that showed the bright orange colour on the reverse colonies, this was calculated by:

$$CFUg^{-1}=\frac{\left(\left(\left(colony\;count\;per\;100\;\mu L\times 10\right)\times reciprocal\;dilution\;factor\right)\times volume\;of\;initial\;suspension\;\left(mL\right)\right)}{weight\;of\;soil\;(g)}$$

### 2.5. Identification of Toxigenic Isolates

All isolates with the appearance of belonging to *Aspergillus* were transferred onto AFPA and CCA and incubated for 5–7 days. Toxigenic strains were identified by a fluorescence under UV light.

### 2.6. DNA Extraction and PCR

DNA was extracted from fungal isolates using the DNeasy Plant Mini Kit (Qiagen: Melbourne, Australia). The PCR primers for *A. flavus* and *A. parasiticus* were obtained using primers designed by Leharanger et al. [4]. The final PCR mix volume was 25 μL, containing: 12.5 μL of Q5^®^ High-Fidelity 2X Master Mix, 1.5 μL each of forward and reverse primers (Table 2), 2 μL of DNA, and 8 μL of H_2_O. The optimal PCR conditions were as follows: 95 °C for 2 min and 40 cycles of 95 °C for 30 s, 59 °C or 54 °C for 30 s, 72 °C for 1 min, finalised by one cycle of 72 °C for 5 min.

### 2.7. HPLC Analysis

HPLC analyses conducted were recorded by PCA, Australia. PCA uses a combination of the AOAC methods for aflatoxin in peanuts (AOAC 1990, Method 968.22 and AOAC Method 980.201). A Waters^TM^ (Sydney, Australia) HPLC instrument was used, with Model 515 pump, Model 717 autosampler and Model 474 fluorescence detector. The extraction and emission wavelengths were 365 nm and 455 nm, respectively, and data acquisition was achieved using a Model 746 Data Module(Waters^TM^ Sydney, Ausralia). The chromatographic separations were conducted on a Nova-Pak^TM^ Phenyl radial compression column supplied by Waters (USA), at a column temperature of 45 °C. Flow rate was 2.0 mL min^−1^ with a mobile phase composition of 20% (*v*/*v*) tetrahydrofuran (THF) in ultra-pure water. Post-column derivatization was achieved by using a saturated solution of iodine in water at a flow rate of 0.9 mL min^−1^ at a temperature of 70 °C. The Limit of Detection was 0.002 to 0.005 ppb, and the Limit of Quantification was 0.10 to 0.25 ppb.

## 3. Results and Discussion

### 3.1. Aflatoxin Component Variants from HPLC Results

**Figure 2:**
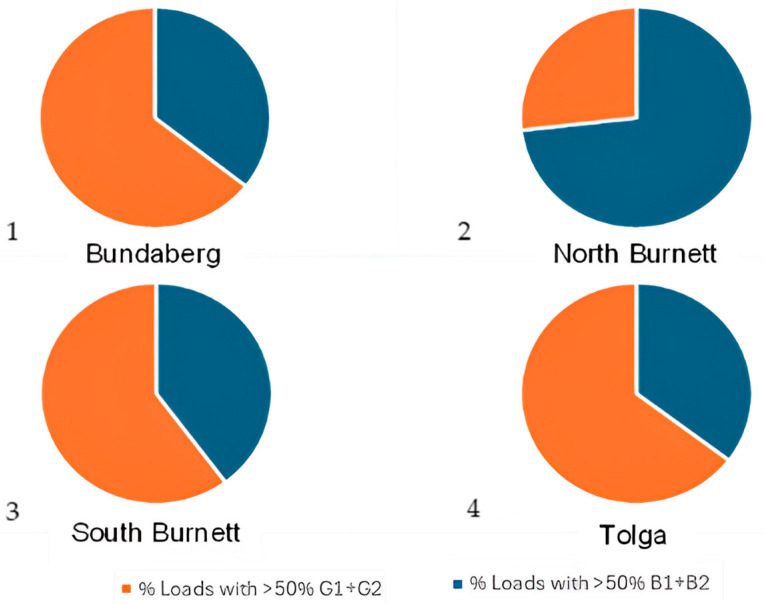
Percentage variation in aflatoxin component variants from HPLC data from 2020 to 2024 for (**1**) Bundaberg, (**2**) North Burnett, (**3**) South Burnett, and (**4**) Tolga.

The historic HPLC data provided by PCA showed that the variation percentage levels of aflatoxin component variants fluctuate by season and location. The Bundaberg, Tolga, and South Burnett regions all showed a higher percentage of aflatoxin B when compared with aflatoxin G over a four-year period (Figure 2).

**Figure 3:**
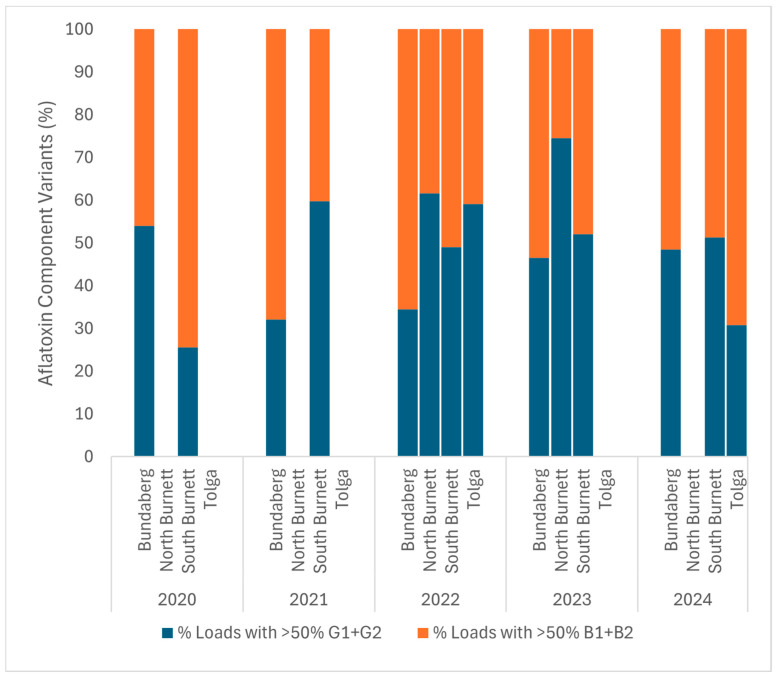
Percentage of aflatoxin component variants across peanut-growing regions and years. In blue is the percentage of peanut loads with over 50% of AFG_1_ and AFG_2_ found and in orange is the percentage of peanut loads with over 50% of AFB_1_ and AFB_2_ for each peanut load from 2020 to 2024 at the PCA facility.

The Bundaberg region had a higher percentage of aflatoxin G in all years except 2020, where aflatoxin B was the dominant aflatoxin. For all years, with positive aflatoxin incidences, North Burnett had a higher percentage of aflatoxin B. South Burnett showed a higher percentage of aflatoxin G in 2020 and a higher incidence of aflatoxin B in 2021. In all other years, South Burnett recorded a similar percentage of aflatoxin B and aflatoxin G. For 2022, the Tolga region recorded a higher percentage of aflatoxin B and a higher percentage of aflatoxin G for 2024 (Figure 3). Not all peanut-growing regions had a positive incidence of aflatoxin across all years. Bundaberg and South Burnett recorded positive incidences of aflatoxin for the years 2020–2024, with North Burnett only recording positive incidences in 2022 and 2023, and Tolga recorded higher in 2022 and 2024 (Table 3, Figure 3).

**Figure 4:**
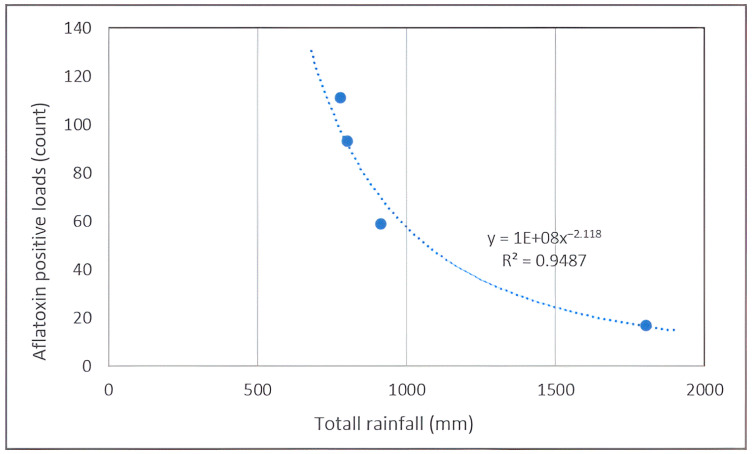
Relationship between total rainfall in different regions and peanut aflatoxin incidences.

End-of-season drought is often attributed to an increase in aflatoxin contamination. When peanuts during the growing season experience adequate rainfall, aflatoxin contamination is generally limited [5]. This is shown in regional rainfall and aflatoxin incidents across QLD: as the volume of rainfall increases the number of aflatoxin-positive loads decreases (Table 3, Figure 4).

### 3.2. Identification of A. flavus and A. parasiticus from Soil Samples

**Figure 5:**
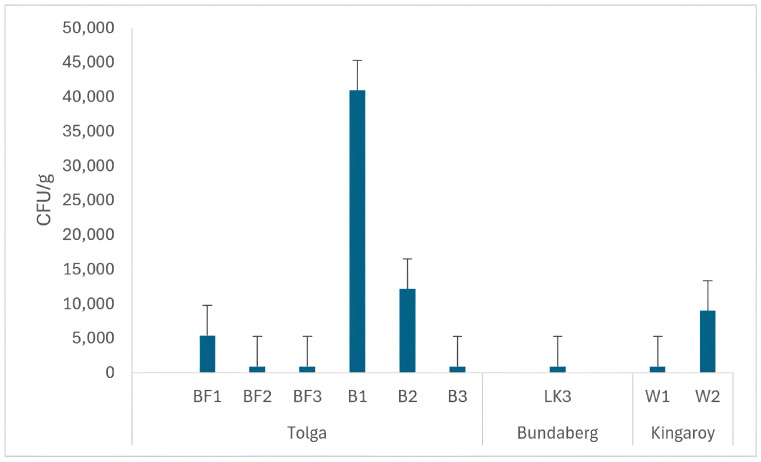
Population densities of filamentous fungi across all regions with positive incidences. Error bars show standard error.

Densities, presented as colony-forming units (CFU/g) of aflatoxin-producing *Aspergillus* species, varied across all regions, with the Tolga region being particularly high, with a total of 61,200 CFU/g across the two sample locations (Figure 5), whereas the Bundaberg region had the lowest density of all regions with only 900 CFU/g from one sample location. Aflatoxin-producing *Aspergillus* species were identified from Tolga, Bundaberg, and South Burnett soils. The highest number of positive samples was recorded from Tolga with 127 colonies. Bundaberg and South Burnett had only three and two colonies, respectively (Table 4).

**Figure 6:**
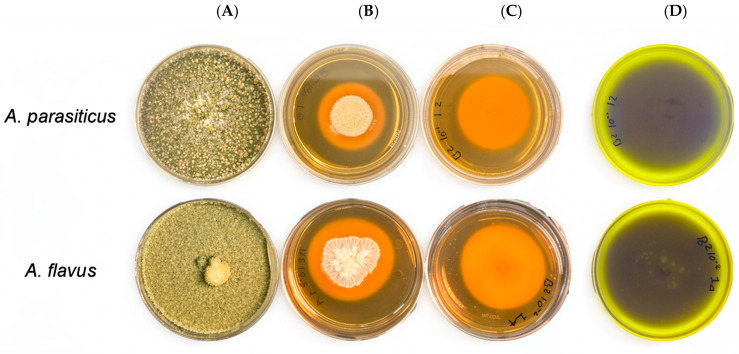
*A. parasiticus* and *A. flavus* isolates on (**A**): CCA, (**B**): AFPA, (**C**): AFPA reverse colony and (**D**): CCA under UV.

Aflatoxin-producing *Aspergillus* species were identified by the characteristic orange reverse colonies when grown on AFPA media (Figure 6). All isolates were transferred to CCA and produced yellow-green and olive conidia, and after seven days of incubation, fluoresced under UV light. The CCA cream agar was first developed to detect aflatoxin production of *A. flavus* and *A. parasiticus* isolates [6]. The fluorescence of isolates on the CCA suggests production of aflatoxin; however, this method has been shown to produce false positives or false negative results [7]. Norlia et al. [8] demonstrated a high correlation with positive fluorescence on CCA and HPLC results for the presence of aflatoxin, whereas other authors have found a low correlation between positive fluorescence and HPLC results [7,9,10]. This method of identifying aflatoxin production of *Aspergillus* isolates is a useful technique when time and resources are limited; however, further analysis is required to accurately confirm if aflatoxin production is present. As the majority of *A. parasiticus* isolates produce aflatoxins [11] and most *Aspergillus* isolates identified were *A. parasiticus* (Table 4), the likelihood of aflatoxins being produced by these isolates is significant.

Identification of *A. flavus* and *A. parasiticus* species was performed using PCR. From a total of 127 individual colonies, 123 were identified as *A. parasiticus* (Table 4). The *A. flavus* colonies were only found in the Tolga region, with a total of four colonies. *Aspergillus flavus* is said to be the dominant aflatoxin-producing *Aspergillus* species in Brazil [12], USA [13], Egypt [14], Kenya [15], China [16], Vietnam [17], Nigeria [18], Iran [19], and in most countries [20]; however, this was not shown in this study for Australia.

The *Aspergillus flavus* and *parasiticus* agar (AFPA) that was initially used is a specialised, selective, and differential culture medium designed for the rapid identification and enumeration of *Aspergillus* species, particularly those in the section Flavi (e.g., *A. flavus*, *A. parasiticus*, *A. oryzae*). While AFPA is highly effective at identifying species within the section Flavi, it is typically used in conjunction with other mycological techniques, such as microscopic examination of conidiophores and vesicles, to achieve full identification. For this reason, a quick and easy PCR method with species-specific primers was used. Although this produces shorter amplicons, it effectively confirms the identities of *Aspergillus flavus* and *A. parasiticus* based on the results from AFPA plating. The use of species-specific primers is both more time- and cost-effective compared to multi-locus DNA sequencing.

Previous work undertaken in Australia [21] claimed that *A. parasiticus* is the dominant aflatoxin-producing *Aspergillus* species in Australian peanut fields, but their work is unpublished. The predominance of *A. parasiticus* isolates found in peanut fields in this study supports these claims. *Aspergillus flavus* is said to be ubiquitous in soil under variable climate conditions, with favourable temperatures of 25 °C to 33 °C, but can grow at temperatures of 30 °C to 40 °C [22]. *Aspergillus parasiticus* is often identified in peanut fields in higher percentages compared with other soil samples [13]. The soil samples in this study were taken from fields that previously grew peanuts; therefore, a higher level of *A. parasiticus* was to be expected. The absence of any *A. flavus* or *A. parasiticus* from the North Burnett region could have been caused by several reasons. The *A. flavus* and *A. parasiticus* isolates were first identified from soil samples on AFPA media by orange reverse colonies. This orange colouring is produced by a reaction with aspergillic acid and the ferric ammonium citrate in the AFPA media. Previous studies [23,24] demonstrate that aspergillic acid is not present in all strains of aflatoxin-producing *A. flavus* isolates. Other work [25] also demonstrates that not all soil samples contain *A. flavus* isolates. The absence of any *Aspergillus* isolates from the North Burnett region could be due to either from species not containing any aspergillic acid, *A. flavus* and *A. parasiticus* species absent from the fields sampled or sampling error. However, there were no aflatoxin-positive peanut samples identified from HPLC results for the peanut-growing season of 2023–2024 from North Burnett (Figure 3).

Populations of *Aspergillus* species have been shown to vary by season, by the dominant vegetation compatibility groups (VCGs) within populations, and by the crop cycles [13,26,27]. Not only do *Aspergillus* populations vary in soil but the proportion of atoxigenic strains varies among countries [22] and mycotoxin production capabilities vary among fields [13]. The selective process that maintains aflatoxigenic and non-aflatoxigenic colonies is unknown [28]. It has been shown that aflatoxigenic strains have lower fitness compared to non-aflatoxigenic strains, possibly attributed to the metabolic cost of mycotoxin production [29]. All *A. flavus* and *A. parasiticus* isolates identified in this study were considered to be aflatoxigenic, based on the presence of fluorescence on CCA (Table 4). Most *A. parasiticus* isolates are also found to be aflatoxigenic [30]; therefore, the presence of fluorescence of the *A. parasiticus* isolates on the CCA strongly indicates that these isolates likely are aflatoxigenic. However, to confirm the presence of aflatoxins in these isolates, further analysis is essential. As all sampling locations had peanuts harvested before sampling, the presence of only aflatoxigenic isolates could be due to selection pressure. To fully understand the *Aspergillus* species across these regions, sampling from non-cropped soil and/or native forests would be required.

## 4. Conclusions

This research evaluated historic HPLC results for variations in aflatoxin component variants from aflatoxin-positive peanut samples collected from peanut harvests during the period 2020–2024. It was found that the dominant aflatoxin component variant was aflatoxin G for peanut-growing seasons 2020–2024 for the Bundaberg, South Burnett, and Tolga regions. The North Burnett region had higher incidences of aflatoxin B. Only the Bundaberg and South Burnett regions had positive aflatoxin samples for each year, with 24 and 40% of all samples, respectively. This research identified the aflatoxin-producing *Aspergillus* species across the four major peanut-growing regions of QLD. *Aspergillus parasiticus* was found to be the dominant *Aspergillus* species in the fields sampled, with 97% (123 of 127) colonies found to be *A. parasiticus.* Only 3% (4 of 127) colonies of *A. flavus* were recorded in the soil sampled from the Tolga region. *Aspergillus* species were not found in the soil samples from the North Burnett region for this sampling event. This could be due to several reasons: sampling error, *Aspergillus* species not producing aspergillic acid leading to colonies not producing the reverse orange colour on AFPA that was used for sampling, or that *Aspergillus* species were not present in the soil sampled. All the *Aspergillus* colonies identified from the soil may or may not be aflatoxigenic; therefore, future studies are recommended for confirming the aflatoxigenic nature of *Aspergillus* species from peanut soil. To produce a more comprehensive identification of *Aspergillus* species in Australian peanuts, soils sampling of native/un-cropped soils is also required, along with further identification of *Aspergillus* species within cropped soil.

## Figures and Tables

**Table 1:** Environmental and climate data from sampling locations. Yearly and Peanut Season (Nov–May) rainfall averages are 10-year averages (2014–2024).

Location	Sample No.	Elevation (m asl)	Climate	Yearly Average		Peanut Season Average		Soil Type
				Rainfall (mm)	Temp. (°C)	Rainfall (mm)	Temp. (°C)	
Tolga	3	594	Tropical	1617.7	23.4–30.8	1446.1	29.3–30.8	Clay and Sandy
Bundaberg	4	31	Subtropical	855.7	22.3–30.4	635.6	29.7–30.4	Clay and Sandy
North Burnet	3	122	Subtropical	711.6	22.9–33.7	526.7	32.8–33.7	Clay
South Burnett	3	434	Subtropical	66.8	19.6–30.9	484.9	29.9–30.9	Clay

Note: All data retrieved from the Bureau of Meteorology [2,3].

**Table 2:** List of primer sequences for identification of *A. flavus* and *A. parasiticus*.

Species	Primers	Sequence (5′-3′)	Aplicon Size (bp)
*Aspergillus flavus*	AfcamF	AATTTTATCCAGTTTCTGTTCGATC	255
	AfcamR	AGGAACTCTATTTGAACATTAACAG	
*Aspergillus parasiticus*	ApcamF	TGGCCGCCATAAGTTTATCAA	183
	ApcamR	CCATTGTTGTCGGCGTCAA	

Note: Obtained from Leharanger et al. [4].

**Table 3:** Count of aflatoxin-positive (>15 ppb) peanut loads from HPLC data (figures in parentheses refer to total samples) and total rainfall (mm) for the peanut-growing regions for 2020–2024.

Crop Year	Bundaberg		North Burnett		South Burnett		Tolga		Year Average	
	Aflatoxin Count	Rainfall (mm)	Aflatoxin Count	Rainfall (mm)	Aflatoxin Count	Rainfall (mm)	Aflatoxin Count	Rainfall (mm)	Aflatoxin Count	Rainfall (mm)
2020	5 (46)	655	-	547	0 (7)	622	-	1288	5 (53)	778
2021	6 (41)	931	1 (23)	735	15 (38)	983	-	1655	22 (102)	1076
2022	15 (79)	1281	86 (103)	1039	60 (137)	917	6 (14)	1475	167 (333)	1178
2023	16 (55)	662	19 (28)	581	18 (50)	481	-	2401	49 (133)	1031
2024	19 (29)	1032	-	995	8 (11)	995	5 (9)	2200	32 (49)	1306
REGION AVERAGE	61 (250)	912	106 (154)	779	97 (243)	800	11 (23)	1804	275 (670)	1074

Note: Data obtained from the Australian Bureau of Meteorology [2].

**Table 4:** Population count of *Aspergillus* isolates grown on AFPA media, percentage of toxigenic isolates, and aflatoxin component variant variation from the 2023–2024 season.

Region	*A. flavus*	*A. parasiticus*	*A. Flavus/A. parasiticus* Ratio	Toxigenic/Non-Toxigenic Ratio %	Loads > 50% AFB_1_ and AFB_2_	Loads > 50% AFG_1_ and AFG_2_
Tolga	4	118	3.3%/96.7%	100%/0%	30.82	69.18
Bundaberg	-	3	0%/100%	100%/0%	48.53	51.50
South Burnet	-	2	0%/100%	100%/0%	51.30	48.70
North Burnett	-	-	-	-	-	-

## Data Availability

Data can be made available by contacting the authors.

## Abbreviations

The following abbreviations are used in this manuscript:

QLD	Queensland
PCA	Peanut Company of Australia
HPLC	High Performance Liquid Chromatography
°C	Degrees Celsius
mm	Milli-metre
m	Metre
AFPA	*Aspergillus flavus* and *parasiticus* Agar
psi	Pound per square inch
CCA	Coconut Cream Agar
mL	Milli-litre
g	Gram
µL	Micro-litre
UV	Ultra-Violet
DNA	Deoxyribose Nucleic Acid
PCR	Polymerase Chain Reaction
s	Seconds
min	Minute
BP	Base Paors
CFU	Colony forming units
VCG	Vegetation Compatibility Groups
